# Insulin Resistance Adversely Affect IVF Outcomes in Lean Women Without PCOS

**DOI:** 10.3389/fendo.2021.734638

**Published:** 2021-09-06

**Authors:** Haoyu Wang, Yu Zhang, Xuhui Fang, Joanne Kwak-Kim, Li Wu

**Affiliations:** ^1^Reproductive Medicine Center, Department of Obstetrics and Gynecology, The First Affiliated Hospital of USTC, Division of Life Sciences and Medicine, University of Science and Technology of China, Hefei, China; ^2^Reproductive Medicine and Immunology, Obstetrics and Gynecology, Clinical Sciences Department, Chicago Medical School, Rosalind Franklin University of Medicine and Science, Vernon Hills, IL, United States; ^3^Center for Cancer Cell Biology, Immunology and Infection Diseases, Chicago Medical School, Rosalind Franklin University of Medicine and Science, North Chicago, IL, United States

**Keywords:** insulin resistance, non-PCOS, lean women, IVF outcome, B cell immunity

## Abstract

**Objective:**

To investigate the effects of insulin resistance (IR) on IVF outcomes and a potential underlying mechanism in lean women without PCOS.

**Design:**

A prospective cohort study at the University Clinic.

**Setting:**

IVF center at the University setting.

**Patients:**

A total of 155 lean women (body mass index <25) without PCOS undergoing IVF cycle.

**Intervention:**

Patients were allocated to IR and non-IR groups based on HOMA-M_120_.

**Main Outcome Measure(s):**

IVF outcomes, including egg quality, the percentage of mature oocytes, fertilization rate, blastocyst formation rate, advanced embryo rate, and cumulative live birth rate were investigated. Auto-immune parameters, peripheral blood immunophenotypes, thyroid hormone, homocysteine, and 25-OH-vitamin D_3_ (25-OH-VD_3_) levels were analyzed.

**Results:**

The percentage of mature oocytes and blastocyst formation rate were significantly lower in the IR group as compared with those of the non-IR group (p<0.05, respectively). The proportion of peripheral blood CD19^+^ B cells was significantly higher in the IR group than those of the non-IR group (p<0.05). Homocysteine, 25-OH-VD_3,_ and auto-immune parameters were the same between the two groups.

**Conclusion:**

In lean infertile women without PCOS, IR is associated with the decreased percentage of mature eggs and poor embryo quality in which B cell immunity may play a role.

## Highlights

Lean non-PCOS women with insulin resistance have adverse IVF outcomes with the decreased percentage of mature oocytes and blastocyst formation rate and increased peripheral blood B cell levels.

## Introduction

Insulin resistance (IR) is typically defined as decreased sensitivity or responsiveness to the metabolic action of insulin (1). IR is caused by a primary defect in insulin receptor signaling and a reduced insulin clearance rate, resulting from decreased hepatic insulin extraction (2). It is commonly associated with obesity, hypertension, cardiovascular disease, and typically type 2 diabetes. It is reported that about 50% to 70% of women with PCOS have IR (3). Infertile women with PCOS had higher levels of fasting insulin, and the resultant hyperinsulinemia plays a role in the pathogenesis of reproductive disorders (4–6).

Hyperinsulinemia disrupts the intrafollicular microenvironment during folliculogenesis and reduces the rate of fertilization and embryonic development potential during the natural and ovarian stimulation cycles (7, 8). Insulin signaling in the uterus controls gene expression and glucose utilization and affects the decidualization process to facilitate implantation (9). IR and free androgen index correlate with total ovarian follicle count in non-PCOS women who underwent IVF-ET treatment. It is suggested that the higher level of IR and androgen can positively affect short-term follicle development and benefit responses to exogenous gonadotrophin stimulation while increasing the risk of OHSS in non-PCOS women (10).

There is increasing evidence at the cellular level showing that inflammation is a critical factor for obesity-induced IR. The tissue-resident immune cells, especially adipose-tissue resident cells, play a major role in regulating obesity-induced inflammation. On the other hand, cellular and molecular factors in adipose tissue regulate obesity-induced inflammation and IR (11). Elevated inflammatory cytokines, including interleukin (IL)-17 and IL-6, could cause a subclinical inflammatory state for a prolonged time. Abnormal inflammatory responses decrease glycolipid metabolism, increase IR, and affect ovulation and fertilization, resulting in polycystic ovary syndrome (PCOS) characterized by oligomenorrhea and irregular ovulation (12).

Obesity is a global epidemic related to numerous health concerns, including reproductive disorders. Central obesity is considered an independent risk factor for early miscarriage (13). Obese individuals exhibit increased estrogen concentrations due to aromatase overexpression in the adipose tissue. Consequently, higher estrogen level causes anovulation via the hypothalamus-pituitary-ovary axis (14). On the other hand, weight loss through lifestyle changes or bariatric surgery positively affects hormonal parameters and ovulation rates (15, 16).

Most of the studies found the effect of IR on IVF outcome in women with overweight/obese PCOS, using homeostasis model assessment for insulin resistance (HOMA-IR) as the index to assess IR. The HOMA index was calculated as HOMA = (fasting insulin uIU/ml * fasting glucose mmol/l)/22.5.HOMA-IR has been reported to effectively predict IR in the overweight-obese PCOS population with a cutoff of 2.62 or more (AUC 84.1%) (17). However, HOMA-IR was found not to be reliable or predictable in detecting IR in lean women, who had neither fasting hyperinsulinemia nor increased basal hepatic glucose production (18). Recently, the HOMA-M_120_ was reported as a reliable and straightforward measure of IR for lean European and Asian women with PCOS (17, 19). HOMA-M_120_ was calculated as (post-load 2-hour insulin uIU/ml * post-load 2-hour glucose mmol/l)/22.5. IR was diagnosed when HOMA-M_120_>12.8. Lean women with HOMA-M_120_>12.8 were considered as IR group, and the others were non-IR group (17). Previous studies have focused on the adverse effect of IR on IVF outcome in women with overweight/obese PCOS. IR’s impact on reproductive outcome in lean PCOS has also been reported (20). Therefore, doctors pay more attention to the diagnosis and treatment of IR in obese and non-obese PCOS patients, metformin for example,to improve IVF outcome.It is noteworthy that IR can also occur in infertile women who had a regular menstrual cycle without polycystic ovaries, which has been reported before (10). But there are no researches about whether IR in lean patients without PCOS adversely affects IVF outcomes. Hence, in this study, we determined to investigate IR by HOMA-M_120_ in lean women without PCOS and analyze the IR effect on IVF outcomes and related cellular and endocrine factors.

## Materials and Methods

### Study Population

This was a prospective observational study done at Reproductive Medicine Center, Department of Obstetrics and Gynecology, the First Affiliated Hospital of University of Science and Technology of China (USTC), Division of Life Sciences and Medicine, USTC, Hefei, Anhui, China, from May 2016 to July 2018. Ethics approval was given by the Ethical Committee of the First Affiliated Hospital of USTC, and all study candidates signed the informed consent form prior to entering the study.

Inclusion criteria were couples with only tubal factor infertility, women’s age from 18 to 40 years old and all underwent a standard long-protocol agonist IVF cycle as the first treatment cycle. Tubal etiology was diagnosed if hysterosalpingography or laparoscopy showed evidence of bilateral tubal obstruction.

Exclusion criteria were women with polycystic ovarian syndrome (PCOS) according to the Rotterdam criteria, high BMI (>25), male infertility, and other endocrine and systemic diseases(including type 1 or 2 diabetes), without familial diabetes history. Patients undergoing ICSI for fertilization failure were also excluded.

**Figure 1:**
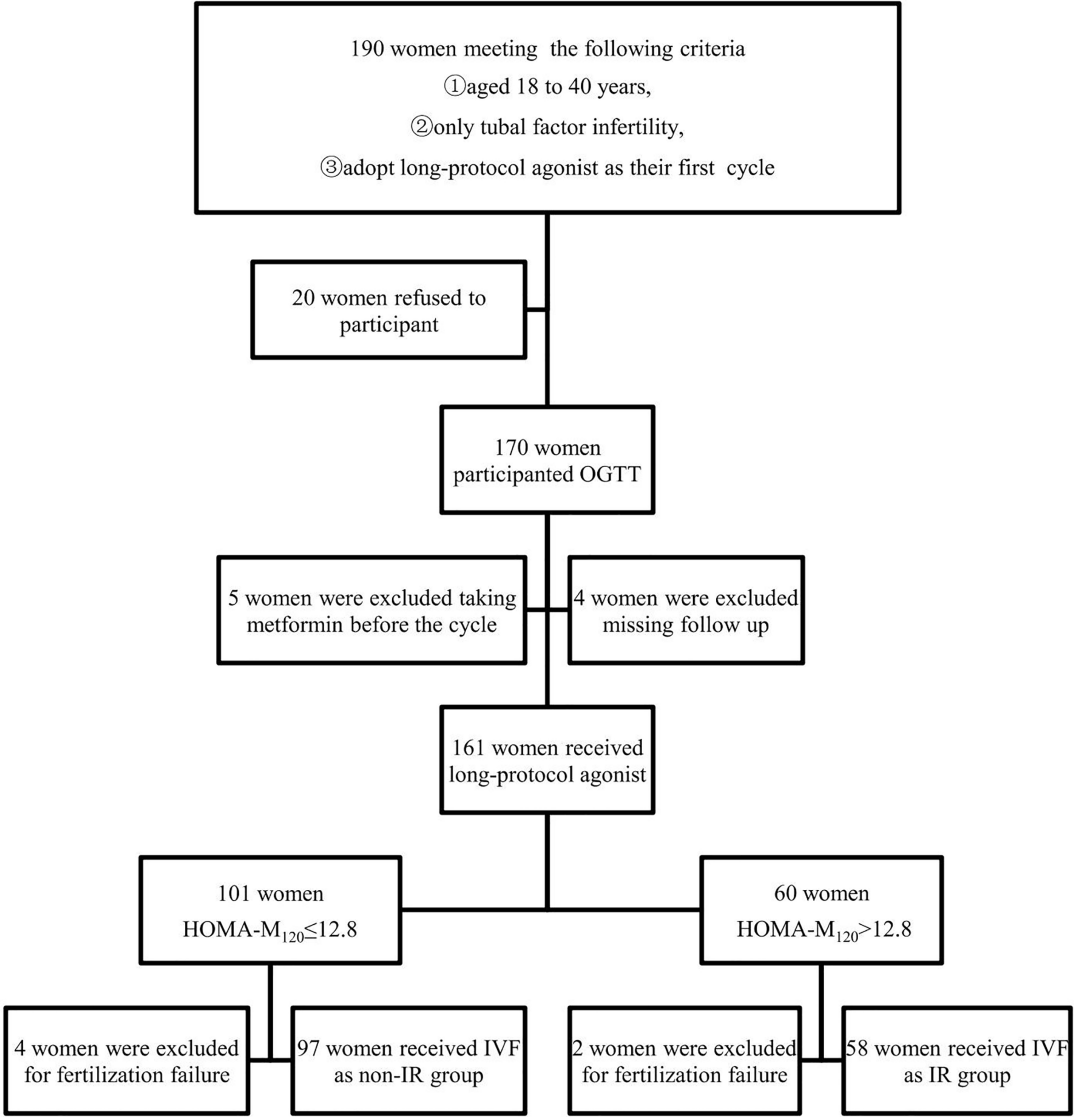
Flow diagram of literature search.

A total of 190 women were asked to participant, but only 170 women agreed to participant and were subjected to an oral glucose tolerance test (OGTT) before the IVF cycle. 5 participants were excluded from the study for taking metformin before the cycle which is known to affect glucose metabolism and insulin sensitivity. Four cases were lost during the follow-up. 6 women were excluded for fertilization failure.Finally, a total of 155 lean infertile women were included in the study. The flow chart is shown in **Figure 1**.

Before the IVF cycle, the BMI, hormonal, biochemical, immunological, and ultrasound parameters were investigated. All underwent a standard long-protocol agonist IVF cycle. Based on the BMI of patients, 0.9-1.5mg Triptorelin was initiated on day 21 to achieve adequate ovarian suppression. After 14 days, pituitary desensitization was checked by estradiol (E_2_) level and B ultrasound scan. Once the criteria for desensitization were satisfied (E 2 ≤ 50  pg/ml, diameter of follicle≤ 5 mm and endometrial thickness ≤ 5 mm), Patients was daily injected 150-225 IU of recombinant FSH (Gonal-F^®^, Merck Serono, Switzerland). Injection of recombinant hCG (Ovitrelle^®^, Merck Serono, Switzerland) 250μg were given when diameter of 2-3 follicle ≥17 mm or at least diameter of one follicle ≥18 mm. Oocytes retrievals were performed 36 h after hCG administration. All patients received conventional IVF as fertilization method regardless of their ages.

During the 2 hour OGTT, fasting blood glucose (FBG), 2-h blood glucose (2-h BG), and 2-h insulin levels were measured. HOMA-M_120_ was used to assess IR in this study, which was calculated as (post-load 2-hour insulin uIU/ml x post-load 2-hour glucose mmol/l)/22.5. IR was diagnosed when HOMA-M_120_>12.8. Lean women with HOMA-M_120_>12.8 were considered as IR group (n=58), and the others were non-IR group (n=97) (17). OHSS has been classified on severity (mild, moderate, severe, critical) according to RCOG classification, a guideline with the title” The Management of Ovarian Hyperstimulation Syndrome” published in 2016.The percentage of mature oocytes, blastocyst formation rate, pregnancy rate, implantation rate, chemical pregnancy rate, abortion, and delivery rates were analyzed. Good-quality embryos mean embryos that reached 6 to 8-cell stage with cytoplasmic fragmentation occupying less than 10% of the embryo surface and had equal size blastomeres. Cumulative live birth was defined as all subsequent embryo transfers during a single oocyte retrieval cycle within 18 months of treatment, including both fresh and frozen-thawed embryo.

### Hormonal Evaluation

All baseline blood sampling including E_2_, follicle-stimulating hormone (FSH), luteinizing hormone (LH), total testosterone (T) and prolactin (PRL) were done during cycle day 2-4 of the menstrual cycle. The oral glucose tolerance test (OGTT) was performed at the same time after overnight fasting and the administration of an oral hypertonic glucose solution (75g). Serum glucose and insulin were measured at 0 and 120 min. Body mass index (BMI) was calculated according to the formula, weight/height^2^. Serum E_2_, FSH, LH, T and PRL were measured employing commercial RIA kits (bioMErieux, Charbonnirres les Bains, France).The serum glucose level was determined by an automatic analyzer employing an enzymatic-colorimetric assay (Gemon, Lindau, Germany). Insulin level was measured by RIA using a commercial kit (CIC bio international, Gif-sur-Yvette, France), and homocysteine (HCY) level was determined using an enzyme conversion immunoassay kit (Axis-Shield, Dundee, UK). The 25-OH-VD_3_ level was measured using the liquid chromatography/tandem mass spectrometry (LC/MS/MS) 155 method at the reference laboratory. Thyroid-stimulating hormone (TSH) and free thyroxine (FT4) were measured by electrochemiluminescence immunoassay (Roche, Germany). The cut-off values for the reference range was 0.4–4.0 mIU/L for TSH and 11–25pmol/L for FT4 (21). Subclinical hypothyroidism was determined when TSH was 2.5-4.0 mIU/L with normal FT4 levels (22).

### Autoantibodies

Antiphospholipid antibodies (APA) and anti-β2GP1 were tested by enzyme-linked immunosorbent assay (ELISA). Anti-nuclear antibodies (ANA) were performed by indirect immunofluorescence using a commercially available kit (Immunoconcepts, Sacramento, CA, USA). Anti-thyroglobulin antibody (ATG) and anti-thyroperoxidase (Anti-TPO) were tested by ELISA (Inova Diagnostic, San Diego, CA, USA).

### Peripheral Blood Immunophenotype

Peripheral blood immune effectors were analyzed by the flow cytometry analysis. Briefly, whole blood samples were labeled using fluorochrome-conjugated monoclonal antibodies (mAb) against CD45-PC5, CD3-FITC, and CD56-PE, or CD45-PC5 and CD19-FITC (Beckman Coulter, Fullerton, CA, USA), and samples were analyzed by a FC 500 flow cytometer using CXP software (Beckman Coulter). Lymphocytes were gated based on side scatter characteristics and CD45 expression. Within lymphocytes, T cells were identified as CD3^+^ cells, NK cells as CD3^-^CD16^+^CD56^+^ cells, and B cells as CD19^+^ cells.

### Statistical Analysis

Statistical analyses were performed using the Statistical Package for the Social Sciences software (SPSS 19, Armonk, NY, USA). Student’s t-test and chi-square test were applied to determine the differences between the means or the distributions of the study groups. Results were presented as mean ± SD for each group, and p-value <0.05 was considered to be significant.

## Results

### Endocrine Profiles

Based on the OGTT and HOMA-M_120_ index, 58 women had IR (37.4%), and 97 women had normal insulin sensitivity (62.6%). Women with IR had a significantly higher incidence of subclinical hypothyroidism (37.93% *vs.* 20.62%, p=0.019). However, age, BMI, antral follicle count, and baseline hormone levels, including FSH, LH, E2, PRL, and T, were similar between IR and non-IR groups. As expected, FBG, 2h BG, FI, 2-h insulin, HOMA-IR were significantly different between the two groups. There were no differences in HCY (7.15 ± 2.67 *vs.* 7.26 ± 2.28, p=0.814) and 25-OH-VD_3_ (18.86 ± 5.40 *vs.* 18.93 ± 8.30, p=0.957) levels in women with IR as compared with those of the non-IR group (**Table 1**).

**Table 1:** Clinical characteristics and endocrine profiles of insulin resistant (IR) and non-insulin resistant infertile women (Non-IR).

	Non-IR (lean) (n = 97)	IR (lean) (n = 58)	P-value
Age (years)	31.43 ± 4.56	30.67 ± 4.65	0.320
Antral follicle number	12.60 ± 6.05	14.43 ± 6.99	0.070
BMI (kg/m^2^)	21.43 ± 1.96	22.07 ± 1.70	0.239
FSH (U/L)	7.93 ± 3.09	7.25 ± 2.87	0.175
LH (U/L)	4.92 ± 3.42	4.74 ± 2.87	0.740
E2 (pg/ml)	49.43 ± 26.92	43.73 ± 31.25	0.232
PRL (ng/ml)	14.46 ± 6.56	15.21 ± 9.03	0.551
Testosterone (ng/ml)	0.45 ± 0.26	0.47 ± 0.36	0.645
FBG (mmol/L)	4.89 ± 0.41	5.11 ± 0.64	0.014^a^
2-h BG (mmol/L)	5.32 ± 0.90	6.88 ± 1.68	0.006^b^
FI (mU/L)	60.17 ± 27.80	97.09 ± 58.41	0.000^b^
2-h Insulin (mU/L)	174.08 ± 83.28	545.17 ± 230.38	0.000^b^
HOMA-IR	1.90 ± 0.93	3.23 ± 2.38	0.000^b^
HOMA-M_120_	6.09 ± 3.27	21.85 ± 8.45	0.000^b^
Sc-Hypothyroidism (%)	20.62	37.93	0.019^a^
HCY	7.26 ± 2.28	7.15 ± 2.67	0.814
25-OH-VD3	18.93 ± 8.30	18.86 ± 5.40	0.957

BMI, Body mass index; FSH, follicle stimulating hormone; LH, luteinizing hormone; E2, estradiol; PRL, prolactin; FBG, fasting blood glucose;2-h BG, 2 hour blood glucose; FI, fasting insulin; HOMA-IR, homeostasis- model assessment of insulin resistance; HOMA-M_120_ was calculated as (post-load 2-hour insulin uIU/ml x post-load 2-hour glucose mmol/l)/22.5.Sc-Hypothyroidism, subclinical hypothyroidism.

P < 0.05 was considered statistically significant; ^a^P < 0.05, ^b^P < 0.01.

### Immune Parameters

**Figure 2:**
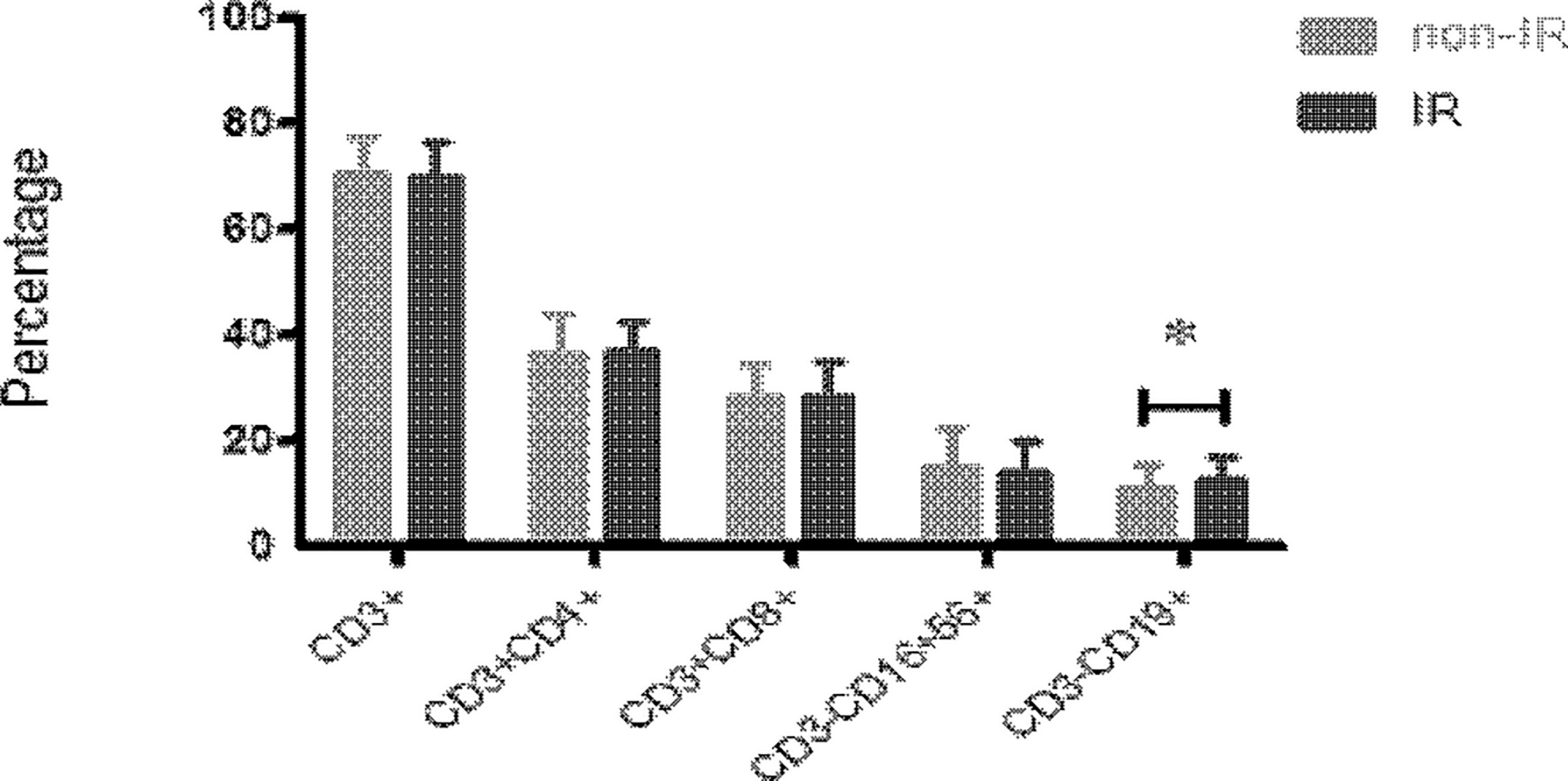
The peripheral blood immunophenotypes of immune effectors, including CD3+, CD3+CD4+, CD3+CD8+, CD3+CD16+CD56+ and CD3+CD19+ cells, were analyzed in the samples of 58 women with IR and 97 women without IR in lean women (16<BMI<25). The proportion of peripheral blood CD19^+^ B cells was significantly higher in lean patients with IR as compared with those without IR. Data are represented as the mean ± SD. Significance was determined using the two-tailed Student t-test. *P < 0.05.

There was no significant difference in auto-immune parameters between IR and non-IR groups, including ATA, ATG, APA, ANA, and a-β2GP1(p>0.05, respectively) (**Table 2**). The proportion of peripheral blood CD19^+^ B cells in women with IR (12.73 ± 4.37%) was significantly higher than that of women with non-IR groups (11.03 ± 4.37%, p=0.032) (**Figure 2**). However, T (70.14 ± 6.35% *vs.* 70.79 ± 6.66%, p=0.581) and NK cell (14.32 ± 5.92% *vs.* 15.13 ± 7.38%, p=0.513) populations in the peripheral blood were not different between the two groups.

**Table 2:** Auto-immune parameters between insulin resistant (IR) and non-insulin resistant (Non- IR) infertile women.

Autoantibodies	Non-IR (lean)	IR (lean)	P-value
ATA/ATG, n (%)	15 (22.06)	9 (18.00)	0.588
APA, n (%)	0 (0)	0 (0)	–
ANA, n (%)	18 (19.78)	11 (20.75)	0.888
anti-β2GP1, n (%)	5 (5.62)	2 (4.00)	0.675

ATA/ATG, anti-thyroid antibody/Anti-thyroglobulin; APA, any IgG or IgM antibodies to phospholipids; ANA, anti-nuclear antibody.

### IVF Outcome

In the IR group, the duration of induction (13.83 ± 3.06 *vs.* 12.70 ± 3.26, p=0.035),the percentage of mature oocytes per oocytes retrieved (85.17 ± 15.16% *vs.* 90.53 ± 12.91%, p=0.02), and freezable blastocysts per residual embryos (39.15% *vs.* 46.46%, p=0.023) were significantly different between the two study groups. However, there were no differences in the number of retrieved oocytes (14.28 ± 9.72 *vs.* 12.13 ± 7.82, p=0.135), the incidence of Ovarian Hyperstimulation Syndrome (20.69% *vs.*16.49%, p=0.511,the number of mature oocytes (12.19 ± 8.93 *vs.* 10.96 ± 7.10, p=0.347), the number of fertilized oocytes (9.98 ± 8.15 *vs.* 8.93 ± 6.54, p=0.221), fertilization rate (81.90% *vs.* 81.46%, p=0.820), the percentage of fresh ET cycles in total ET cycles(39.62% *vs.* 45.98%, p=0.462) and the percentage of good-quality embryos per cleaved embryos (56.50% *vs.* 56.97%, p=0.869). In non-IR group, 14 patients were diagnosed with mild OHSS and 2 patients with moderate OHSS. In IR group, 10 patients were diagnosed with mild OHSS and 2 patients with moderate OHSS. No severe or critical OHSS happened in either non-IR group or IR group. The small sample size limited the stratification analysis. But we found there was no difference in the incidence of mild OHSS (20.83% *vs.*16.86%, p=0.64).

In the first ET cycle, chemical pregnancy rate(41.51% *vs.* 28.74%, p=0.121),pregnancy rate (32.08% *vs.* 26.44%, p=0.474), chemical pregnancy loss rate(22.73% *vs.* 8%, p=0.157),clinical pregnancy loss rate (35.29% *vs.* 13.67%, p=0.111) and pre-term births rate (18.18% *vs.* 10.53%, p=0.552) between IR and non-IR group were not different.Besides, an ectopic pregnancy occurred in non-IR group in the first ET cycle. Moreover, cumulative live birth rate per oocyte retrieval cycle (46.55% *vs.* 50.52%, p=0.633) was not different between the two study groups (**Table 3**).

**Table 3:** Oocyte and embryological data after IVF-ET treatment between insulin resistant (IR) and non-insulin resistant (Non-IR) infertile women.

	Non-IR (n = 97)	IR (n = 58)	P-value
Duration of induction (days)	12.70 ± 3.26	13.83 ± 3.06	0.035^a^
No. of retrieved oocytes	12.13 ± 7.82	14.28 ± 9.72	0.135
No. of mature oocytes	10.96 ± 7.10	12.19 ± 8.93	0.347
No. of fertilized oocytes	8.93 ± 6.54	9.98 ± 8.15	0.221
% of mature oocytes per oocyte retrieved	90.53 ± 12.91	85.17 ± 15.16	0.02^a^
% of fertilized oocytes among the mature oocytes	81.46 (866/1063)	81.90 (579/707)	0.820
% of good-quality embryos per cleaved embryos	56.97 (429/753)	56.50 (291/515)	0.869
% of freezable blastocysts per residual embryos	46.46 (269/579)	39.15 (157/401)	0.023^a^
% of fresh ET cycles in 1st ET cycles	45.98 (40/87)	39.62 (21/53)	0.462
% of chemical pregnancy in 1st ET	28.74 (25/87)	41.51 (22/53)	0.121
% of pregnancy in 1st ET	26.44 (23/87)	32.08 (17/53)	0.474
% of chemical pregnancy loss in 1st ET	8 (2/25)	22.73 (5/22)	0.157
% of clinical pregnancy loss in 1st ET	17.39 (4/23)	35.29 (6/17)	0.196
% of pre-term births in 1st ET	10.53 (2/19)	18.18 (2/11)	0.552
Cumulative live birth rate,%	50.52 (49/97)	46.55 (27/58)	0.633

P < 0.05 was considered statistically significant, ^a^P < 0.05.

## Discussion

In this study, we found that IR is associated with a slower response to ovulation induction, poor oocyte maturation, and decreased proportion of freezable embryos in lean and non-PCOS women who underwent IVF treatment, with increased B cell levels as compared to non-IR women.

In this study, the prevalence of subclinical hypothyroidism was significantly higher in the IR group than the non-IR group among lean women without PCOS, which is consistent with the previous study demonstrating that the HOMA-M_120_ index was significantly increased in women with subclinical hypothyroidism (23). There are several possible mechanisms to explain the observed relation between low-normal thyroid function and IR. Insulin might influence thyrotropin-releasing hormone (TRH) and TSH when modulating glycemic status (24), and subclinical hypothyroidism is associated with decreased insulin sensitivity and glucose tolerance, partially due to a decreased insulin ability to increase glucose utilization mainly in the muscle (25).

We have demonstrated that the retrieved number of oocytes was higher in the IR group than the non-IR group, although the difference did not reach a significant level, which is consistent with the previous study (10). Insulin promotes primordial to primary follicle transition (26). Additionally, the response of granulosa cells to FSH during the gonadotropin-dependent stage of folliculogenesis can be enhanced by insulin growth factors (27). Therefore, in the IVF setting, the risk of developing multifolliculogenesis or ovarian hyperstimulation syndrome to exogenous gonadotropin stimulation is higher in IR than non-IR patients (28).

In our study, the percentage of mature oocytes per oocytes retrieved and the percentage of freezable blastocysts per residual embryos were significantly lower than those of women in the non-IR group. Previous study found insulin could stimulate theca cell androgen production, elevating serum free testosterone levels, so hyperinsulinemia will increase the local production of androgens (29). It is proved that high level of androgen can interfere with fertilization and cleavage rates of in vitro–matured oocytes, which decrease the number of mature oocytes and blastocyst formation rate (30). In our study, the percentage of mature oocytes per oocytes retrieved and the percentage of freezable blastocysts per residual embryos were significantly lower than those of women in the non-IR group. This is consistence with previous study. But the direct mechanisms of hyperinsulinemia adverse mature oocytes and blastocyst need further study.

As metformin often induces side effects, new integrative strategies have been proposed to treat insulin resistance, such as the use of inositols. Myo-inositol (MYO) and d-chiro-inositol (DCI) are two inositol stereoisomers in humans. MYO is the precursor of inositol triphosphate, a second messenger that regulates thyroid-stimulating hormone (TSH) and FSH as well as insulin. Several preliminary studies suggest that a deficiency of D-chiro-inositol (DCI) containing IPG might be at the basis of insulin resistance (31). In fact Genazzani et al. reported that MYO administration can not only decrease fasting insulin plasma levels in obese patients (32) but also improve insulin sensitivity in non-obese PCOS patients (33).

Finally, in contrast to the non-IR group, the IR group had a significantly higher level of peripheral blood B lymphocytes. Since there is no difference in autoimmunity between the IR and non-IR groups, increased B cells in the IR group may reflect the expansion of the functional subset of B cells. Previous study found B cells can worsen glucose tolerance by production of IgG antibodies and activation of proinflammatory macrophages and T cells in mice model. Depletion of B cells in mice can ameliorate glucose tolerance and fasting insulin. However, the return of B cells will exert their detrimental effects on glucose tolerance again. These results indicates B cells play a role in insulin resistance (34). In our study, we found B cells were increased in IR group, this was consistence with previous studies. Further studies are required to confirm the mechanism of the speculation.

Previously, we reported that women with low vitamin D levels had higher peripheral blood B-cell proportion and T helper/T cytotoxic cell ratios than those of normal vitamin D (35). In this study, vitamin D levels were not different between the two study groups. Maybe the IR in lean women with non-PCOS didn’t have vitamin D deficiency tendency.

The study is limited since the underlying mechanism of higher B lymphocyte count in the peripheral blood of women with IR was not explored further and auto-antibodies to insulin and its receptors were not investigated. Since the genetic study of the embryos were not made in all cases, IR and genetic abnormalities of the embryos were not thoroughly investigated.

Currently, the independent role of IR in IVF outcomes is not defined well (30, 36). Our study demonstrated the association between IR and adverse IVF outcomes in the lean non-PCO population. Possible use of anti-glycemic agent during the IVF cycle should be investigated in the future in women with IR without obesity or PCOS.

## Data Availability Statement

The original contributions presented in the study are included in the article/supplementary material. Further inquiries can be directed to the corresponding authors.

## Ethics Statement

The studies involving human participants were reviewed and approved by the Ethical Committee of the First Affiliated Hospital of USTC. The patients/participants provided their written informed consent to participate in this study. Written informed consent was obtained from the individual(s) for the publication of any potentially identifiable images or data included in this article.

## Author Contributions

All authors qualify for authorship by contributing substantially to this article. HW and LW were responsible for study design and conception, data analysis, the first draft of the article, review, and approval of revisions, and the final article. JK-K interpreted the data and revised the manuscript. XF and YZ were responsible for recruitment, data collection, and article review. All authors contributed to the article and approved the submitted version.

## Funding

This study was supported by the National Natural Science Foundation (No. 81601353, No.82071650).

## Conflict of Interest

The authors declare that the research was conducted in the absence of any commercial or financial relationships that could be construed as a potential conflict of interest.

## Publisher’s Note

All claims expressed in this article are solely those of the authors and do not necessarily represent those of their affiliated organizations, or those of the publisher, the editors and the reviewers. Any product that may be evaluated in this article, or claim that may be made by its manufacturer, is not guaranteed or endorsed by the publisher.
